# Efficacy Study of Propolis Eutectic Extract in Gel Formulations for the Treatment of Bacterial Skin Diseases in Dogs

**DOI:** 10.3390/ani15101434

**Published:** 2025-05-15

**Authors:** Dovilė Svetikienė, Monika Jokubaite, Gintaras Zamokas, Lina Babickaite, Rita Šiugždiniene, Kristina Ramanauskiene

**Affiliations:** 1 Department of Dr. L. Kriauceliunas Small Animal Clinic, Faculty of Veterinary Medicine, Lithuanian University of Health Sciences, Tilzes Str. 18, LT-47181 Kaunas, Lithuania; gintaras.zamokas@lsmu.lt (G.Z.); lina.babickaite@lsmu.lt (L.B.); 2 Department of Drug Chemistry, Faculty of Pharmacy, Lithuanian University of Health Sciences, Sukileliai Avenue 13, LT-50162 Kaunas, Lithuania; monika.jokubaite@lsmu.lt; 3 Institute of Microbiology and Virology, Faculty of Veterinary Medicine, Veterinary Academy, Lithuanian University of Health Sciences, Tilzės Str. 18, LT-47181 Kaunas, Lithuania; rita.siugzdiniene@lsmu.lt; 4 Department of Clinical Pharmacy, Faculty of Pharmacy, Lithuanian University of Health Sciences, Sukileliai Avenue 13, LT-50162 Kaunas, Lithuania

**Keywords:** pyoderma, topical medication, hydrogels, oleogels, bigels, propolis, DES extract

## Abstract

Skin infections are common in veterinary practice and are often treated with topical agents. Superficial pyoderma (superficial bacterial folliculitis) is a common cause of skin disease in dogs and a reason for treatment, most caused by *Staphylococcus* spp. strains. The frequent use of antibiotics contributes to the emergence of resistant bacterial strains, making antimicrobial resistance (AMR) one of the most important threats to human and animal health. For this reason, active natural compounds are increasingly being explored as alternative therapies. To contribute to the development of effective treatments for bacterial infectious diseases, researchers are looking for new antimicrobial agents. Topical drug action has many advantages as it avoids systemic reactions and ensures that the active substance reaches the site of the lesion directly. This study aimed to develop gelled dosage forms with propolis extract and to evaluate their antibacterial activity and the release of the active substances. Hydrogels, oleogels, and bigels enriched with eutectic propolis extract were produced. Deep eutectic solvents (DESs) were chosen as an effective tool to extract the active compounds of propolis and to improve their penetration into the skin. The pH values of the semi-solid pharmaceutical forms tested ranged from 3.3 to 6.4. Using modified Franz-type diffusion cells, the release of phenolic compounds from gels, oleogels, and bigels was assessed and quantified spectrophotometrically using the Folin–Ciocalteu method. The highest amount of active compounds was released from the hydrogels, while the lowest amount was released from the castor oil-based oleogel. The study used clinical and reference strains of bacteria. The antimicrobial activity of the gelled dosage forms with propolis extract was tested against six pathogenic bacterial species (*S. aureus*, *S. agalactiae*, *B. cereus*, *E. faecalis*, *E. coli*, *Ps. aeruginosa*) and one pathogenic fungus (*C. albicans*). The study’s results suggest that the propolis extract obtained by DES has significant antibacterial activity and is a promising component in skin formulations for the treatment of bacterial infections.

## Simple Summary

Skin infections are common in veterinary medicine and are often treated with topical preparations. Superficial bacterial folliculitis (SBF) is most commonly caused by *Staphylococcus* spp., and frequent antibiotic use may contribute to the development of resistant strains. Antimicrobial resistance (AMR) is a major threat to human and animal health and natural compounds with antibacterial properties, such as propolis, are being explored as alternative therapies to combat it. In this study, different types of gels with propolis extract were developed, including hydrogels, oleogels, and bigels. These gels help to deliver active compounds directly to the site of infection. The study investigated the antibacterial activity of propolis extracts. The results showed that propolis extract demonstrated promising antibacterial activity, making it a suitable active ingredient for the treatment of bacterial skin infections.

## 1. Introduction

The dog’s skin is the largest organ in the body and is constantly exposed to various internal and external stressors. It also has an important function as an immunological barrier that requires specific nutrients. Therefore, even small changes in diet or imbalances in the immune system can significantly affect the condition of the skin and coat [1]. The most common canine skin problems are related to nutritional deficiencies, hormonal disorders, infections, allergens, and other external factors that can cause inflammatory reactions. Flea infestations, bacterial infections, and allergic conditions like atopic dermatitis are among the most frequently observed disorders. Additionally, genetic and environmental factors significantly contribute to their onset and progression [2,3,4,5]. Superficial bacterial folliculitis (SBF), or superficial pyoderma, is a bacterial infection confined to the superficial part of the hair follicle and is a common condition diagnosed in pets. Bacteria can cause infection due to local trauma, scratches, contamination from inadequate care, seborrhea, parasite infestations, hormonal factors, local irritants, or allergies [6]. In dogs, superficial bacterial folliculitis (SBF) is the most prevalent type of pyoderma and represents a primary reason for antimicrobial use in small animal veterinary practice [7,8]. The predominant pathogen causing superficial pyoderma is *Staphylococcus pseudintermedius*, a commensal bacterium found in mucous membranes and on the skin surface [9,10]. Other strains of *Staphylococcus* spp., and less frequently Gram-negative bacteria, are also typical causative agents of pyoderma [11]. It is much more usual in dogs and cats than in humans due to differences in the physiological and anatomical properties of the skin. Compared to humans, the stratum corneum of dogs is thinner, contains fewer intracellular lipids, and has a higher pH [11,12,13]. Given the limited availability of antimicrobial agents for the treatment of skin diseases in veterinary medicine, it is important to find new alternatives that are easy to apply, non-irritating to the skin, and can be easily rinsed off without sticking to the animal’s coat. Topical treatment is widely used for a range of diseases because it avoids passage through the gastrointestinal tract, prevents gastrointestinal irritation, and delivers active compounds directly to the lesion, minimizing unnecessary adverse reactions. For a bioactive composition to be therapeutically effective, it must enable the drug to penetrate the stratum corneum barrier when needed to reach its target site. The condition of the skin barrier, the selected drug delivery system, the formulation components, accurate disease identification, and proper dosage planning all impact the effectiveness of topically administered drugs. Some of the most widely used pharmaceutical forms in dermatology are semi-solid preparations such as ointments, creams, and gels. Proper selection of the pharmaceutical form and excipients ensures good application to the skin and localization at the site of action. Formulations intended for the treatment of the epidermis routinely use bases without penetrating components. When the drug needs to reach the deeper layers of the skin and the bloodstream, excipients or mixtures of excipients are selected to increase membrane permeability [12]. The formulations developed must have the right pH value, viscosity, sensory properties, and stability during storage and use. In this study, the production of hydrophilic gels, oleogels, and bigels with eutectic propolis extract was chosen. Non-ethanol extracts could be an excellent alternative to propolis ethanol extracts, provided that the solvent chosen for their production effectively separates the active substances from the raw material. This has been confirmed by experimental studies by our research group using DES in the production of propolis extracts. The researchers demonstrated that the use of deep eutectic solvents (DESs) for the extraction of propolis is an innovative method that solves the problems associated with traditional solvents such as ethanol. The uniqueness of DES lies in its ability to adjust polarity, allowing efficient extraction of the bioactive compounds in propolis, by green chemistry principles. This technology has the potential to prevent skin irritation. In our earlier research, propolis extracts obtained using DES exhibited a statistically significant increase in the total active compound content compared to extracts made with poloxamer (P407) [14]. In addition, studies have confirmed that DES, including mixtures of choline chloride and propylene glycol, are effective and viable alternatives [15]. As structural systems, biogels (such as hydrogels, oleogels, and bigels) function as delivery vehicles for bioactive agents. Oleogels and hydrogels are two important solid-form compositions often used in cosmetic and medical applications. The combination of these two materials produces a so-called bigel, which has distinctive properties that allow for more efficient delivery of both hydrophilic (water-soluble) and lipophilic (lipid-soluble) active substances [16]. Systems that are viscous polymer liquids at room temperature but gel at physiological temperatures within the living organism, forming a viscoelastic composition, are promising. Thermosensitive hydrogels with the sol–gel transition at physiological body temperature are widely used in medicine due to their ability to gel at the point of use and to ensure the localization of drugs, thereby reducing systemic toxicity. Thermosensitive hydrogels provide high adhesion to the mucosal surfaces of natural tissues, localized delivery, and a reduction in the dose of drug administered. Hydrogels are manufactured from synthetic (e.g., poloxamers, poly-N-isopropylacrylamides) or natural (e.g., chitosan, cellulose) polymers. These biomaterials are often used in the treatment of acute and chronic wounds due to their ability to promote tissue regeneration and to fill in uneven wound areas, and further research is focusing on improving their viscosity, mechanical strength, and stability, with a view to broader clinical applications [17]. The thermoreversible gelling agent poloxamer was chosen for hydrogel production as a safe and efficient carrier for the effective transport of therapeutic agents to the target cells [18]. For the oleogel, vegetable oils were chosen as the oil phase, and yellow beeswax was used as the gelling agent. Oleogels are excellent carriers for lipophilic bioactive compounds [19]. Bigels have attractive properties such as better spreadability, enhanced drug penetration, and higher hydration of the stratum corneum, and are a potential pharmaceutical form for the treatment and prevention of skin diseases in veterinary medicine. Currently, increasing attention is being paid to the search for sources of active compounds of natural origin to avoid antimicrobial resistance (AMR) and adapt them to animal health problems [20]. In addition to oral administration, many plant extracts have anti-inflammatory, antibacterial, and antifungal properties when applied topically. These substances are effectively used in the treatment of various dermatological problems such as canine atopic dermatitis, pruritic dermatitis, pyoderma, otitis externa, infected wounds, and dermatophytosis. Due to these properties, plant extracts are being evaluated as potential tools for the topical treatment of skin conditions [21,22]. One such substance that has been extensively studied by scientists is the bee product propolis. Propolis is a natural substance made up of a complex mixture of resinous materials that bees gather from buds, flowers, and plant secretions, which they combine with their saliva, wax, and pollen [23]. Propolis typically contains about 50% plant resins, 30% waxes, 10% essential and aromatic oils, 5% pollen, and 5% other organic compounds [24,25]. The effectiveness of various types of propolis is closely linked to their intricate chemical composition, which can differ based on factors like the season, the location where the plant resin is gathered, and the species of bees involved. The chemical composition of propolis is closely related to and dependent on the raw plant material collected by bees in different regions of the world, which can lead to differences in its chemical composition. Propolis varies in chemical composition from region to region and even from place to place, with more than 300 substances identified to date, including phenolic acids, flavonoids, esters, diterpenes, sesquiterpenes, lignans, aromatic aldehydes, aromatic alcohols, fatty acids, vitamins, and minerals [26]. In temperate climates, the main source of beeswax is the buds of poplar trees. Research has shown that the main components of the buds are phenolic acids and flavonoids, which are responsible for the diverse pharmacological effects of propolis, including antibacterial, antioxidant, antifungal, antitumor, analgesic, anti-inflammatory, and therapeutic effects. Different species of poplar trees are characterized by different amounts and compositions of active compounds. Extracts of balsam poplar buds were found to contain a higher content of active compounds compared to extracts of black poplar and propolis. *p*-coumaric acid is predominant in these buds and propolis. The studies provided new data on the chemical composition and bioactivity of Lithuanian poplar buds, as well as more information on the similarities and differences in chemical composition and bioactivity between propolis and poplar buds. According to the literature, Lithuanian, Polish, and Latvian propolis is classified as poplar propolis. Propolis extracts from the Baltic region share a similar chemical composition and consistent biological effects. Our previous research confirms that *p*-coumaric acid is the dominant compound in the chemical profile of propolis extracts from this area [27,28]. Unfortunately, the application of propolis in veterinary medicine is limited. The scientific literature provides evidence of the beneficial effects of propolis in wound healing. Propolis accelerates wound healing, promotes tissue regeneration, and limits scar formation [29,30]. Studies conducted by Ashraf M. Abu-Seida reported that the use of propolis increased wound shrinkage and reduced healing time. Due to its antibacterial, antifungal, anti-inflammatory, and antioxidant properties, propolis is a natural and promising agent that can assist in managing infectious skin problems in animals [20]. Ethanolic extracts of propolis are widely used in pharmacology. However, as a solvent, ethanol can have toxic and irritating effects on the skin; therefore, searching for alternative solvents is necessary [31,32,33]. This study aims to model gelled delivery systems with eutectic propolis extract and evaluate the influence of the substrate as a carrier on the release of active compounds and antibacterial activity.

## 2. Materials and Methods

### 2.1. Materials

Raw Lithuanian propolis (“Bičių korys”, Vilnius, Lithuania); beeswax (“Medaus namai”, Antazaves village, Lithuania); choline chloride (Sigma-Aldrich, St. Louis, MO, USA); lactic acid (Sigma-Aldrich, USA); olive oil (Sigma-Aldrich, Steinheim, Germany); castor oil (Sigma-Aldrich, Steinheim, Germany); poloxamer P407 (Fagron, St. Paul, MN, USA); purified water; NaOH (Erba Lachema, Brno, Czech Republic); laboratory electronic balance (Kern PBS/PBJ, Kern, Germany); analytical balance (Scaltec SBC 31, Scaltec Instruments GmbH, Göttingen, Germany); water bath (Harry Gestigkeit GmbH, Düsseldorf, Germany); pH meter 766 with electrode Knick SE 104 N (Knick Elektronische Meßgeräte GmbH & Co., Berlin, Germany); viscometer (Vibro viscometer SV-10, A&D Company Ltd., Tokyo, Japan); magnetic stirrer with heating surface (IKAMAG C-MAG HS7, IKA-Werke GmbH & Co. KG, Staufen im Breisgau, Germany); spectrophotometer (Agilent 8453 UV-Vis, Agilent Technologies, Santa Clara, CA, USA).

#### Preparation of Propolis DES Extract

A 30% propolis extract was prepared using DES. The eutectic solvent was created by adding appropriate amounts of the starting materials and stirring at a specific temperature, with choline chloride, lactic acid, and purified water in a ratio of 2:1:2. Extraction was carried out by maceration. The macerates were stored for 12 days in dark glass bottles at 21 ± 1 °C, with intermittent stirring of the macerate contents [34]. After 12 days, all extracts were stirred using a magnetic stirrer at 40 ± 1 °C, 620 rpm, for 60 min. The extracts were then cooled and stored for 24 h at 6 ± 1 °C. The extracts were filtered through ashless filter paper (retention 8–12 µm, diameter 90 mm, ash content 0.007%).

### 2.2. Modeling of Semi-Solid Dosage Forms

#### 2.2.1. Hydrogel with Propolis DES Extract

The gels were prepared at varying concentrations (13%, 15%, 18%) using poloxamer 407 (P407) as the base. The compositions of the gels are provided in Table 1. The required amount of poloxamer 407 was accurately weighed and mixed with an appropriate volume of purified water. The mixture was stored in a refrigerator at 4 ± 1 °C for 24 h to ensure complete dissolution of the polymer. Once dissolved, the mixture was stirred until a homogeneous semi-solid gel was formed. Subsequently, the appropriate amount of propolis DES extract (10 g extract per 100 g gel) was incorporated into the gel bases. The pH of the gels was adjusted to the desired range of 5.5–6.5 by adding a 10% sodium hydroxide solution (*v*/*v*) and stirring the mixture until a homogeneous mass was obtained.

#### 2.2.2. Oleogels and Bigels with Propolis DES Extract

In Phase I, the oleogel bases were prepared. Vegetable oil was poured into a porcelain saucer, and the required amount of beeswax was weighed and melted in the vegetable oil using a water bath. The mixture was stirred with a glass rod. After a homogeneous mass was formed, the propolis DES extract (10 g extract per 100 g oleogel) was incorporated into the oil phase and mixed until a consistent texture was obtained.

In Phase II, bigels were prepared with a 1:1 ratio of hydrogel to oleogel (G2:OG1; G2:OG2). The appropriate amount of hydrogel base was mixed with an equal amount of oleogel. Propolis DES extract (10 g extract per 100 g bigel) was added to the homogeneous base. All experimental formulations were kept in a refrigerator at a temperature of 5 ± 1 °C. The compositions of the oleogels and bigels are presented in Table 1.

#### 2.2.3. Determination of pH and Microstructure of Semi-Solid Pharmaceutical Preparations

The pH values of the gels, oleogels, and bigels of the semi-solid pharmaceutical preparations produced were determined using a pH meter. The pH of each semi-solid experimental formulation was measured three times. To examine the microstructure of the semi-solid pharmaceutical preparations, a drop of each formulation was placed on a glass slide and covered with a cover slip. The microstructure was evaluated using a light microscope (Olympus BX36, Tokyo, Japan) with a video camera (Olympus DP72, Tokyo, Japan). The image analysis software “CellSensDimension V1.16” was used.

#### 2.2.4. In Vitro Release Study

The release of phenolic compounds from gels, oleogels, and bigels was evaluated using modified Franz-type diffusion cells. Prior to the experiment, the semi-permeable membrane was soaked in purified water for 12 h. The donor phase contained 1.0 g of the semi-solid preparation, while the acceptor phase consisted of a 30% (*v*/*v*) ethanol solution. The test was conducted at 37.0 ± 0.5 °C. To assess the release of phenolic compounds from the gels, samples of the acceptor solution were collected at 6 h. The spectrophotometric Folin–Ciocalteu method was employed according to the publication by Bobinaitė et al., with some modifications [35]. One milliliter of the propolis extract was diluted in 2 mL of 70% (*v*/*v*) ethanol. Then, 0.2 mL of the diluted extract was mixed with 5 mL of 10% Folin–Ciocalteu’s reagent and 4 mL of 7.5% (*v*/*v*) Na_2_CO_3_ solution. The test samples were stored in the dark at room temperature for 60 min. Absorbance was measured at 765 nm. The results are expressed as milligrams of gallic acid equivalent per gram of dry weight (mg GAE/g).

#### 2.2.5. LOD and LOQ

The Limit of Detection (LOD) refers to the smallest quantity of an analyte that can be detected in a sample, while the Limit of Quantitation (LOQ) represents the lowest amount of an analyte that can be accurately quantified with suitable precision and accuracy. Both values are determined using the standard deviation (SD) of the response and the slope, according to the following equations:

LOD = 3.3 × SD/S

LOQ = 10 × SD/S

SD—residual standard deviation

S = slope, which according to the graph is 0.1771

LOD = 0.58 mg/mL

LOQ = 1.77 mg/mL

This means that the lowest detectable concentration is around 0.58 mg/mL, while reliable quantification is possible from 1.77 mg/mL.

#### 2.2.6. Determination of Antimicrobial Activity

The evaluation of in vitro antimicrobial activity was carried out based on previous studies [36]. The bacteriological properties of the experimental formulations were evaluated in vitro by the disk diffusion agar method using Mueller–Hinton agar (Biolife Mueller Hinton Agar II, Milan, Italy), Colombian blood agar (CBA, EO Labs, Bonnybridge, Scotland, UK), and Sabour dextrose agar (Liofilchem, Roseto degli Abruzzi, Italy). Mueller–Hinton and Sabour dextrose agars were prepared to Clinical and Laboratory Standards Institute (CLSI) approved standards and dispensed into 10 cm diameter Petri dishes of ~35 mL each. To cultivate *Streptococcus agalactiae*, Colombian blood agar in Petri dishes was purchased from the manufacturer (CBA, EO Labs, Bonnybridge, Scotland, UK). Clinical (*S. aureus*, *B. cereus*, *E. feacalis*, *E. coli*, *Ps. aeruginosa*, *S. agalactiae*) and reference (*S. aureus* (ATCC 25923), *B. cereus* (ATCC 11778), *E. feacalis* (ATCC 29212), *E. coli* (ATCC 25922), *Ps. aeruginosa* (ATCC27853), *S. agalactiae* (ATCC 13813)) strains were used. Suspensions of 0.5 McFarland density were prepared from the clinical isolates and reference strains. Clinical and reference bacterial strains were obtained from the Lithuanian University of Health Sciences, the Academy of Veterinary Medicine, and the Institute of Microbiology and Virology, from small animals with acute superficial or deep pyoderma, wound infections, and abscesses. A total of 50 µL each of clinical and reference strains of *S. aureus*, *B. cereus*, *E. faecalis*, *E. coli*, and *Ps. aeruginosa* were spread on Müller–Hinton agar, and *S. agalactiae* on blood agar. Additionally, 50 µL each of the clinical and reference *C. albicans* strain suspensions were spread on Sabour dextrose agar. Subsequently, wells (7 mm in diameter) were made in a Petri dish into which 0.1 mL of the test substance was poured. The plates containing the bacteria were kept for 24 h 37 ± 0.5 °C in a thermostat. The plates containing the fungus were stored for 24 h in a thermostatic flask 35 ± 0.5 °C in a thermostat. The in vitro antibacterial and antifungal properties of the semi-solid dosage forms were evaluated after incubation for 24 h in a thermostat. The diameter in millimeters of the sterile zones formed around the wells was calculated. The measurements were made in triplicate. The tests were carried out on G1-G2, OG1, OG2, BG1, and BG2 semi-solid formulations.

To compare the susceptibility of the tested bacterial strains to DES extract and amoxicillin with clavulanic acid, susceptibility to the antibiotic was determined using the Kirby–Bauer disk diffusion method (EUCAST, 2024). Amoxicillin/clavulanic acid (AMC; 30 µg) was used. The susceptibility of *S. aureus* to the amoxicillin/clavulanic acid compound was evaluated by susceptibility to cefoxitin. The results were evaluated according to the EUCAST (European Committee on Antimicrobial Susceptibility Testing) 2024 Guidelines for *Enterobacterales* and *Staphylococcus* spp. *Bacillaceae* susceptibility was determined based on the susceptibility/resistance criteria for *Staphylococcus* spp. as there are no established clinical cut-off values using the disk method. The susceptibility of *Ps. aeruginosa* and *C. albicans* to amoxicillin with clavulanic acid was not investigated because these strains are inherently resistant.

#### 2.2.7. Statistical Analysis

The results are expressed as the mean and standard deviation of three measurements. Differences were considered statistically significant at *p* < 0.05. For independent measurements, the nonparametric Kruskal–Wallis test was used. Statistical analysis and data visualization were performed using GraphPad Prism, version 10.0 (GraphPad Software, San Diego, CA, USA), IBM SPSS Statistics 27 (SPSS Inc., Chicago, IL, USA), and SigmaPlot 13.0 (Systat Software, San Jose, CA, USA) software tools.

## 3. Results

### 3.1. Production of Hydrogels, Oleogels, and Bigels with Propolis DES Extract

The total phenolic compounds in the propolis extract produced were found to be 115 mg/g GAE. The lactic acid in the DES used for the production of the extract gives the extract a pH value of pH < 4. Therefore, the sodium hydroxide solution was used as a pH-adjusting agent in the modeling of the gel formulations. Three hydrogels with different compositions were produced using varying concentrations of poloxomer P407: 13%, 15%, and 18%. The hydrogels, consisting of poloxomer and propolis extract, had a semi-solid consistency, a light brownish color, and a pleasant aroma. In the next step of the study, the oleogel was prepared. A propolis DES extract was added to the oil base containing beeswax. The prepared oleogels were also semi-solid, with a light brownish to yellowish color and a pleasant aroma. In the third stage of the study, the bigels were prepared. The bigels were created in a 1:1 ratio of hydrogel to oleogel. They displayed a semi-solid texture, ranging in color from yellowish to whitish, with a pleasant fragrance. The compositions of the formulations produced are presented in Table 1.

### 3.2. Physicochemical Properties of Semi-Solid Pharmaceutical Preparations

To evaluate the stability of the experimental formulations, it is essential to determine their physicochemical properties. In this study, the pH of the semi-solid pharmaceutical preparations was measured, and their microstructure was analyzed. The results of our microstructural study show that the surface relief of the gel samples (G2 and G3) was almost uniform, and the addition of the propolis extract did not affect the compatibility of the components. The internal structure was homogeneous with no signs of phase separation. However, the structure of gel G1 showed droplets of dispersion, which are likely to contain the propolis extract. These results suggest that the consistency of the gel with propolis extract depends on the amount of poloxamer P407 used for gelation. In the matrix of the oleogels (OG1, OG2), the propolis extract is distributed in the aqueous phase, forming a water-in-oil structure. In the photographs of the bigel compositions (BG1, BG2), the fusion of the oleogel and the gels is observed, and droplets are visible in the structure. The oleogel phase is distributed in the homogeneous gel medium, forming oil droplets. The addition of sodium hydroxide led to the formation of gels in the pH range of 6.0–6.4. The oleogels had a strongly acidic pH value (3.3–3.6). The pH value of the bigel compositions (5.1–5.4) was statistically significantly (*p* < 0.05) lower than that of the gels but higher than that of the oleogels.

### 3.3. In Vitro Release from Semi-Solid Formulations

**Figure 1:**
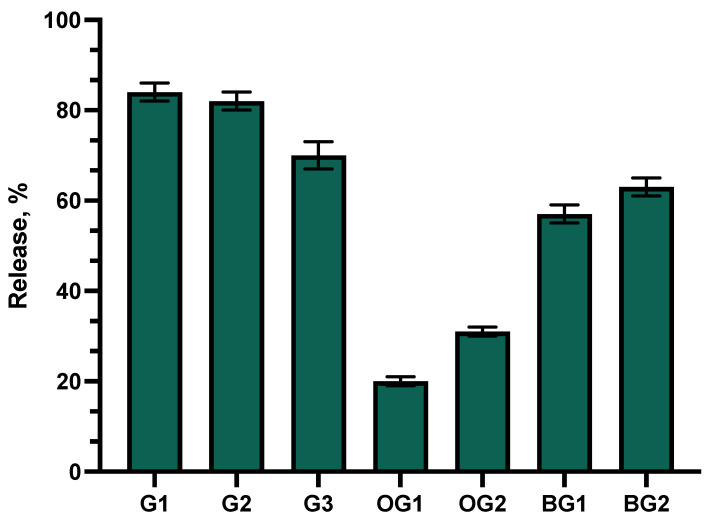
Percentage of total phenolic compound release versus square root of time. Percentage of total phenolic compounds released from G1–G3 gels, OG1–OG2 oleogels, and BG1–BG2 bigels (n = 3, mean ± SD).

An in vitro release test of the formulations was performed. This test evaluates the impact of selected gelling agents on the release of active ingredients from the formulated gels. The results of the test are presented in Figure 1.

The data showed that the highest levels of active compounds were released from gels G1 and G2. Statistically significantly lower amounts of active compounds were released from gel G3 compared to formulations G1 and G2 (*p* < 0.05). After 6 h of testing, similar amounts of phenolic compounds were released from formulations G3 and BG2. Statistically significantly lower amounts of phenolic compounds diffused into the acceptor medium from bigel BG1 compared to formulations G1–G3 and BG2 (*p* < 0.05). Among all the formulations tested, oleogel OG1, based on castor oil, showed the lowest release of active compounds.

### 3.4. Determination of Antimicrobial Activity

The antimicrobial activity evaluation tests showed that oleogels OG1 and OG2 did not inhibit the growth of the bacteria tested.

**Figure 2:**
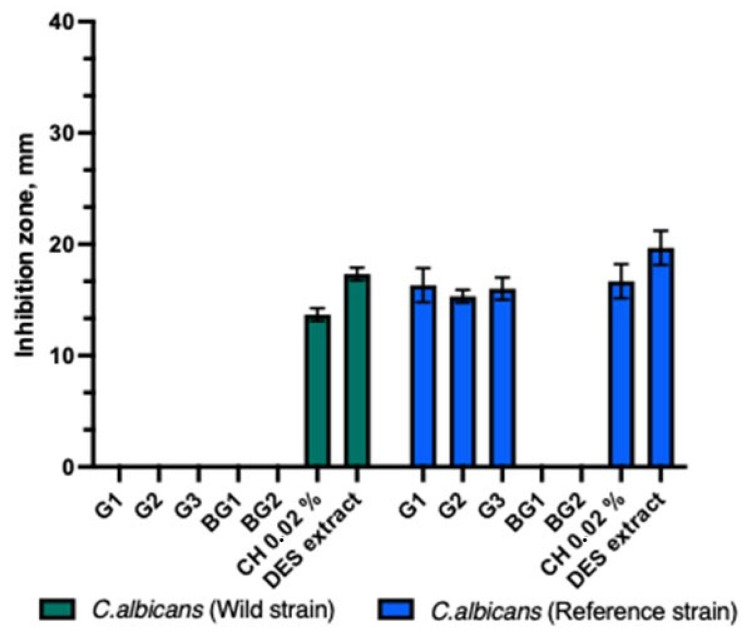
Antifungal activity of propolis gels against *C. albicans*.

The antifungal activity tests with the *C. albicans* strain showed that the test gels (G1-G3) exhibited antifungal activity only against the reference *C. albicans* strain (Figure 2). The control DES propolis extract had a statistically significantly larger inhibitory zone compared to the test gels and the chlorhexidine solution (*p* > 0.05). The other tested formulations did not inhibit the growth of the *C. albicans* strain.

**Figure 3:**
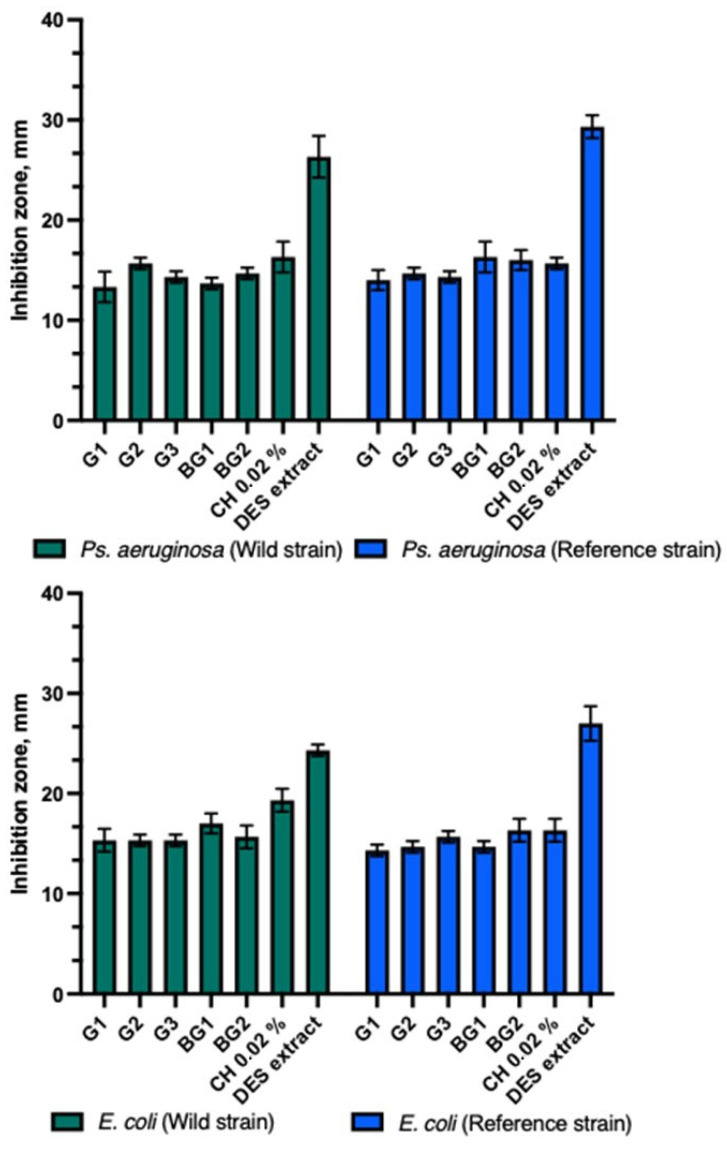
Antibacterial activity of propolis gels and bigels.

The experimental formulations of the gels (G1–G3) and bigels (BG1–BG2) demonstrated similar antimicrobial activity against reference and clinical strains of *Ps. aeruginosa* and *E. coli* bacteria (Figure 3). The DES propolis extract showed a statistically stronger antibacterial inhibitory effect than the tested gels, bigels, and the positive control. A statistically significant difference was also observed (*p* > 0.05). The control group with a 0.02% chlorhexidine solution exhibited similar antibacterial inhibitory effects as the tested gels and bigels.

**Figure 4:**
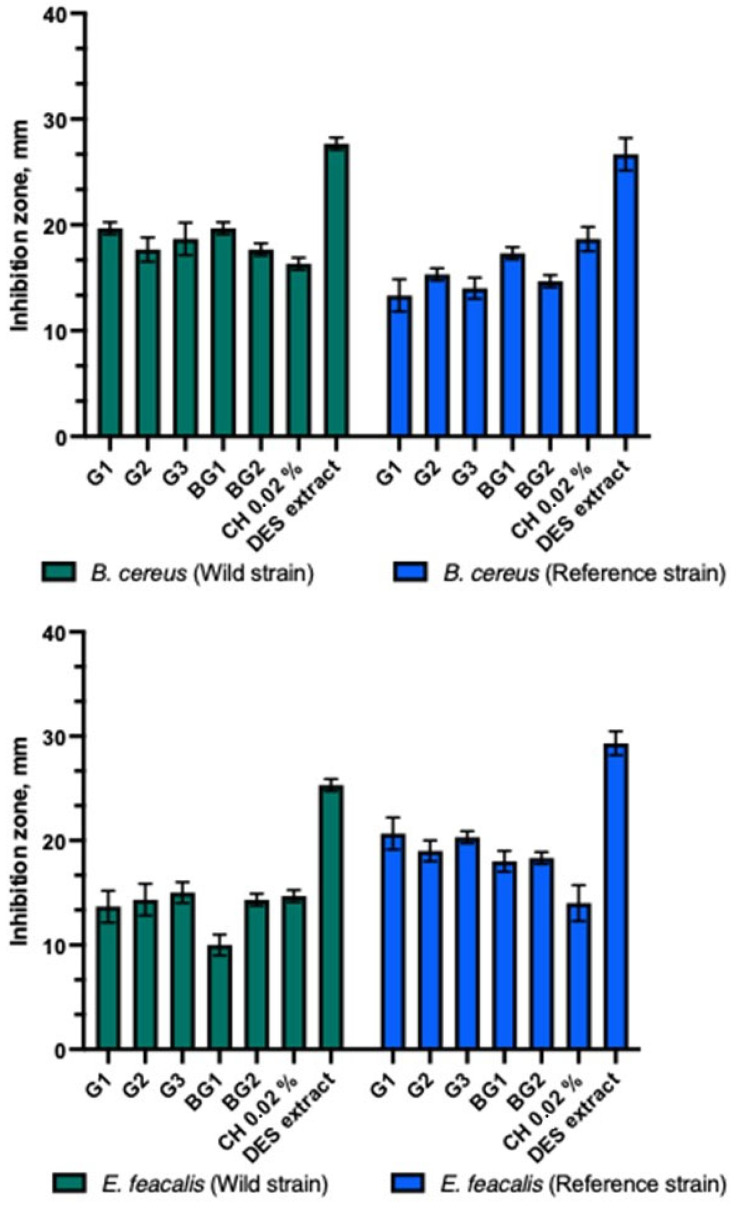

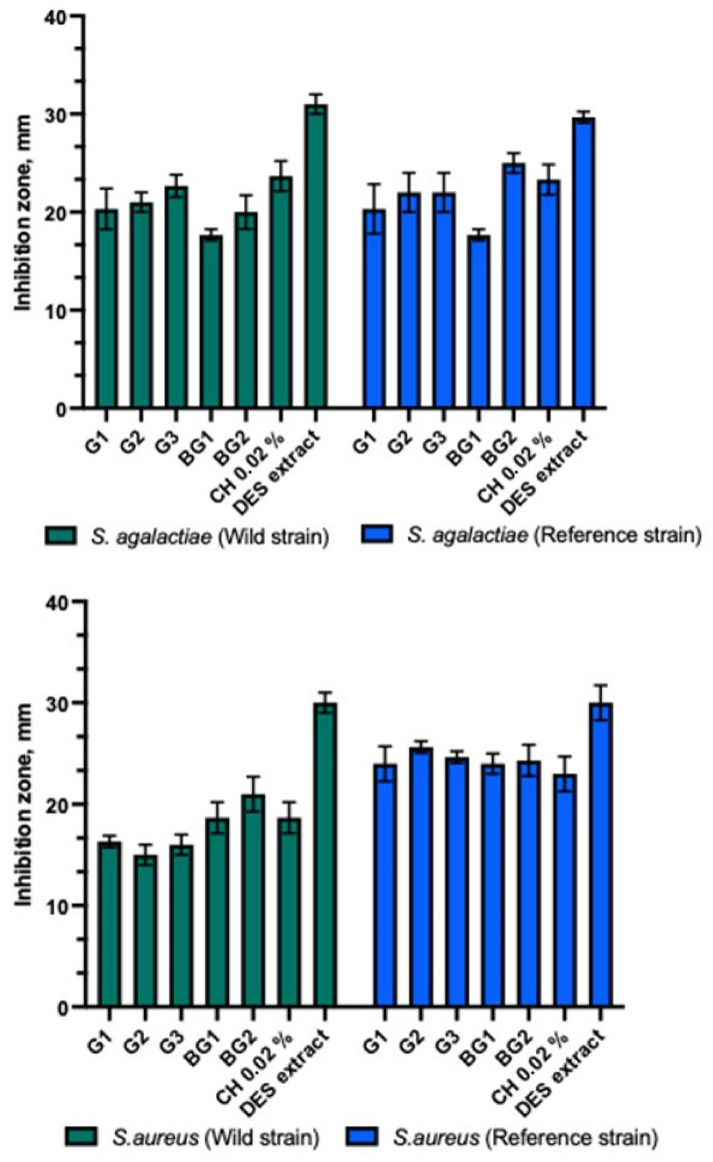
Antibacterial activity of propolis gels and bigels.

The gels and bigels showed antibacterial activity against Gram-positive bacterial strains (Figure 4). The strongest antibacterial inhibitory effect of gels and bigels was observed against the reference *S. aureus* compared to other Gram-positive bacteria tested. A significant difference was found (*p* > 0.05). The gel and bead formulations tested showed similar inhibition zones against clinical and reference *S. agalactiae* strains. Bigel BG2 showed a statistically significantly stronger effect on the clinical *B. cereus* than the reference one (*p* > 0.05). All gels and bigel BG1 showed similar antibacterial activity against clinical and reference bacterial strains. The BG1-BG2 bigels demonstrated a statistically significantly stronger antibacterial inhibitory effect against the reference strain of *E. faecalis* compared to the clinical strain (*p* > 0.05). Both bigels and gels exhibited a stronger effect on the reference *E. faecalis* strain than on the clinical strain. A statistically significant difference was observed between these zones (*p* > 0.05). The DES propolis extract demonstrated a statistically significantly stronger antibacterial inhibitory effect compared to the tested gels, bigels, and the positive control. However, no statistically significant difference was observed (*p* > 0.05). The control group with the 0.02% chlorhexidine solution exhibited a similar antibacterial inhibitory effect as the studied gels and bigels.

**Figure 5:**
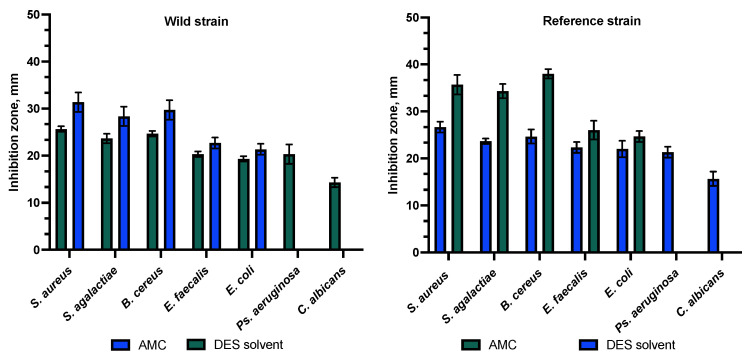
Antibacterial activity to combination of amoxicillin and clavulanic acid (AMC) and DES extract.

As amoxicillin with clavulanic acid is commonly used to treat skin diseases, AMC disks were chosen as a negative control. The study also included an assessment of the susceptibility of the tested bacterial strains to a combination of amoxicillin and clavulanic acid (AMC), except *Ps. aeruginosa*, which is inherently resistant to beta-lactam antibiotics and was not susceptible to AMC. In this study, *S. aureus*, *B. cereus*, *S. agalactiae*, *E. faecalis*, and *E. coli* were found to be susceptible to AMC. As *C. albicans* is a eukaryote, it has not been tested for susceptibility to any antibiotic. The selected eutectic diluent served as a positive control and demonstrated antibacterial and antifungal activity against both the clinical and reference isolates. The DES triplicate exhibited statistically significantly stronger activity against clinical strains of *S. agalactiae*, *E. faecalis*, *E. coli*, *Ps. aeruginosa*, and *C. albicans* compared to AMC. A statistically significant difference was observed (*p* > 0.05). AMC had a statistically significantly stronger effect on *S. aureus* and *B. cereus* clinical strains. A statistically significant difference was observed (*p* > 0.05). DES had a statistically significantly stronger effect on the reference strains of *E. feacalis*, *E. coli*, *Ps. aeruginosa*, and *C. albicans* compared to AMC, but AMC had a statistically significantly stronger impact on the reference strains of *S. aureus*, *S. agalactiae*, and *B. cereus*. A statistically significant difference was observed between the zones (*p* > 0.05). The results indicated that the solvent components of the DES played a significant role in the antibacterial activity of the propolis extracts against the tested bacterial and fungal isolates, as shown in Figure 5.

## 4. Discussion

The emergence and spread of antimicrobial resistance (AMR) present a significant threat to global health systems, making AMR action plans crucial for addressing its components, including the screening of new compounds to discover novel antimicrobials [37]. The resurgence of natural products has been fueled by advances in many different research areas. All of this is leading to the curbing of AMR [38]. To tackle this problem, the focus in recent years has shifted towards studying natural substances that offer newer therapeutic options. From an economic perspective, these are more affordable than conventional therapies and are beneficial in treating subclinical or chronic diseases where conventional antimicrobial treatments may not be effective [39,40]. The treatment of canine dermatological diseases requires a comprehensive approach, requiring a combination of several forms of intervention, such as avoidance of various allergens, immunotherapy, and antimicrobial and anti-inflammatory therapy [41,42]. In addition, studies show that the use of natural substances is relevant in the treatment of canine dermatological diseases. New approaches, including the incorporation of phenolic compounds and herbal extracts, suggest that these substances may help to reduce clinical dermatological signs while ensuring a high quality of life for the animals [43,44,45]. The use of natural substances in veterinary medicine has been the focus of numerous studies aimed at improving animal health and treatment. Several researchers have explored the antimicrobial activity of natural products against canine isolates from the *Staphylococcus* and *Enterobacteriaceae* families. These studies have examined various natural sources, such as herbal extracts, honey products, and bacteriophages [46,47]. Studies in Spain have shown that as many as 70% of patients with dermatological diseases were treated with herbal preparations, highlighting the practical potential of herbal sources [48]. Recent evidence suggests that plant-derived compounds such as oregano essential oil (OEO), olive, and castor oils may have significant antibacterial, antioxidant, and anti-inflammatory effects, paving the way for their use in animal therapy [49,50,51,52]. In recent years, an increasing number of in vivo and in vitro studies have been conducted on the effects of plant extracts, which encourages the consideration of the type of extract and the extraction method used [21,53]. In today’s context, where more sustainable and effective therapeutic solutions are being sought, the study of natural substances opens new avenues toward the integration of innovative deep eutectic solvent (DES) technologies into the formulation of natural compounds. DES allows for improvement in the bioactivity and penetration of active substances into the skin while ensuring their safety and compatibility with epithelial tissue regeneration. This technology is considered promising not only for the treatment of skin diseases but also for the modeling of jellified systems with the extraction of propolis active compounds using DES, which do not adversely affect wound healing, to achieve a more effective penetration of active substances into the skin. Numerous studies have shown that phenolic compounds in oils have anti-inflammatory, neuron-protective, anti-aging, and cell repair properties [54,55,56]. Oils used as components in oleogels and bigels serve as the oil phase, which is capable of forming stable gel structures. The components of DES can be chosen not only to adjust the physicochemical properties of the selected solvents but also to enhance the biological activity of DES extracts [57]. In light of the results of our previous studies [14], it is concluded that the DES propolis extract produced is suitable for incorporation into semi-solid experimental pharmaceutical dosage forms because of its biological properties. Also to model the base with the semi-solid dosage forms, the polymer poloxomer P407 was chosen. Poloxomer ((poly(ethylene oxide-poly(propylene oxide-poly(ethylene oxide) copolymer)) is a polymer frequently used in the production of hydrogels, which has low toxicity and indifference and is suitable for semi-solid formulations due to its thickening, stabilizing properties [58,59]. These properties can enhance the product’s consistency, extend the drug release time, and improve its effectiveness in topical applications. While gels are commonly used in pharmaceutical formulations, they may not always be suitable depending on the skin condition. Gels may fail to provide effective transdermal delivery of the active ingredient and may not offer sufficient skin hydration or nourishment [60,61]. Therefore, in the next phase of the study, oleogels with DES propolis extract were produced. Such systems are widely used in skin formulations [62]. In a later stage of the study, bigels were produced by combining hydrogel and oleogel systems [63]. Bigels consist of both hydrophobic (oleogels) and hydrophilic (hydrogels) components and are a good candidate for delivering both hydrophobic and hydrophilic bioactive materials individually or simultaneously. New scientific discoveries on the use of propolis in veterinary medicine as a natural therapeutic ingredient are highlighted. According to recent studies, propolis added to experimental gels can effectively fight skin infections caused by coagulase-positive *Staphylococcus* spp. in dogs. This is supported by the antimicrobial and anti-inflammatory properties of propolis. This aspect is significant because the potential of propolis as a natural therapeutic agent can be extended to modern innovative products used in veterinary practice. This demonstrates not only the efficacy of propolis but also the technological feasibility of developing functional and long-lasting therapeutic solutions [64]. The results of our microstructural evaluation of the experimental formulations show that the propolis extract additive is distributed in the bases of gels (G1–G3) and oleogels (OG1, OG2). In the study of the experimental compositions of the beads, as can be seen from the photographs of BG1 and BG2, when the different phases interact, a homogeneous structure is formed. The oleogel phase, evenly distributed in the gel medium, forms small oil droplets that become microscopically visible. This means that the propolis extract is also involved in this integration, ensuring a uniform distribution of the active ingredients in the bigel. Toro-Vazquez and other researchers have developed a water–oleogel emulsion based on candelilla wax and MSM, bearing in mind that not only the oleogelator but also candelilla wax can help stabilize water droplets [65]. The structure of the bigel is essential for the efficient transport of active ingredients, especially when aiming for a slow and controlled release from the bigel matrix. Such interactions and homogeneity of the structure may lead to improved penetration of active ingredients through the skin, as described in the study by Ziliaus et al. on the in vitro release of propofol phenolic acids from pause-solid formulations in the human skin in Lithuania [66]. The formation and stability of the beryllium structure are highly dependent on the molecular interactions of the constituents and the solubility of the phases, which is important for pharmaceutical and cosmetic formulations [67]. Our analysis of the results showed that the G1 and G2 gel formulations released the highest amounts of active compounds, with 13% and 15% of poloxomer P407, indicating that these gel formulations are the most effective in ensuring high release of active substances, which could lead to a faster wound healing process. In contrast, the G3 formulation showed a statistically significantly lower release of active compounds, which may be related to the amount of poloxomer P407 used (18%) for gelation, which results in a different gel structure. A lower release of phenolic compounds was observed in the case of the BG1 bigel and oleogels, especially in the OG1 formulation using castor oil. These results suggest that oleogels, especially those made with castor oil, may be suitable for the sustained release of active substances, which is important for the development of slow-acting semi-solid systems and for the contribution to slow healing processes. On the other hand, our studies show that the incorporation of propolis in oleogels can improve the penetration of the drug into the skin due to the water/oil structure and that the distribution of propolis in the aqueous phase in the oleogels can form a suitable matrix for the transport of the active ingredient. Other researchers have shown that the phenolic compounds in propolis can interact with gelling agents that help to stabilize the active compounds in the oleogel matrix. The structure of the oleogel, especially the water-in-oil emulsion type, facilitates better penetration of the bioactive compounds through the skin. The hydrophobic structure of the oleogel helps to increase the solubility of the phenolic compounds of propolis in the lipid layers of the skin, thus improving the transdermal delivery of the active compounds [68]. The study results indicated that the release of active compounds from semi-solid formulations is influenced not only by the drug concentration in the formulation but also by the properties of the carrier and the solubility of the active compounds within the chosen carrier. These findings confirmed the antimicrobial activity of propolis and offered additional insights into the biological properties of propolis extracts and their potential role in combating antimicrobial resistance (AMR) [69,70]. Based on the antimicrobial activity tests carried out with the *C. albicans* strain, it was found that the test gels (G1–G3) showed antimicrobial activity only against the reference *C. albicans* with zone diameters of (15.33 ± 0.58–16.33 ± 1.53 mm). The other formulations tested did not inhibit the growth of *C. albicans*. This result suggests that although propolis may be effective in antimicrobial therapy, the choice of formulations and their components is important to ensure the optimal effect against specific micro-organisms. In an in vitro antimicrobial evaluation study with experimental semi-solid formulations, we observed that the oleogels (OG1, OG2) did not inhibit the growth of isolates of the families *Staphylococcaceae*, *Streptococcaceae*, *Bacillaceae*, *Enterococcaceae*, *Enterobacteriaceae*, *Pseudomonadaceae*, and *Saccharomycetaceae*. This can be explained by the slowest release of active compounds in the in vitro release study. The antibacterial efficacy may also have been influenced by the composition and structure of the oleogels, where the efficacy of the propolis is dependent on the presence of a carrier in the oleogel, which may interfere with the release of propolis compounds. Another possibility for the lack of antibacterial efficacy of the oleogels against the bacterial isolates tested is that the propolis compounds do not dissolve or diffuse out of the oleogel in the same way and therefore do not reach the necessary concentration to have an antibacterial effect. The oleogel may act as a barrier limiting the release of the active components of propolis. Further results showed that the gels (G1, G2, G3) showed antibacterial efficacy against *S. aureus*, *S. agalactiae*, *B. cereus*, *E. feacalis*, *E. coli*, and *Ps. aeruginosa*. The diameters of the zones of inhibition in the clinical strains were (13.33 ± 1.53–22.67 ± 1.15 mm), reference (13.33 ± 1.53–34.33 ± 0.58 mm). Meanwhile, bigels also inhibited the growth of the tested isolates. The diameters of the zones of antibacterial efficacy against *S. aureus*, *S. agalactiae*, *B. cereus*, *E. feacalis*, *E. coli*, and *Ps. aeruginosa* in the preclinical strains were (10.00 ± 1–21.00 ± 1.73 mm), reference (16.00 ± 1–34.00 ± 2 mm). The antibacterial activity of the semi-solid experimental compositions in these studies may be attributed to the more rapid release and diffusion of the propolis into the agar, and therefore the antibacterial activity may have been due to the release of the active ingredients [71]. According to researchers, the efficacy of some antimicrobial agents may be correlated with time rather than with high peak concentrations [72]. Gels and bigels provide a better release of active substances such as propolis extracts compared to oleogels. Water-based gels facilitate the release of water-soluble substances and contact with bacterial cells, resulting in a stronger antibacterial effect. Water-based gels and bigels have better penetration of bacterial cell membranes because water is a good solvent for many antibacterial compounds. This increases the likelihood that the active ingredients in the gel will interact directly with the bacterial membranes, damaging them and causing their death. Gels often allow the active components to be evenly distributed throughout the formulation, which ensures that the active ingredients are continuously exposed over time, resulting in a more potent antibacterial effect [73,74,75,76]. The DES propolis extract demonstrates significant antibacterial activity, with the solvent components of DES contributing substantially to the antibacterial effects against the tested bacterial strains. This makes DES an excellent component for inclusion in skin preparation formulations aimed at treating bacterial infections. The novelty of this study lies in a deeper assessment of the importance of carrier selection, as it was found that a well-selected carrier not only ensures the efficient release of the active compounds of propolis but also enhances its antibacterial activity. Studies show that poloxomer-based gels are particularly promising as carriers, as they guarantee a targeted release of propolis active compounds at the site of infection. These results underline the potential of bigels as an innovative form of treatment, combining an antibacterial effect with the added benefit of skin regeneration.

## 5. Conclusions

The integration of propolis extract into various skin preparation forms, such as gels and bigels, opens up new opportunities for developing advanced dermatological products for the treatment of bacterial infections. The study demonstrates not only the antibacterial efficacy of these formulations but also their additional benefits for skin health, which could represent a significant breakthrough in both veterinary and medical practices. The chosen DES had antibacterial properties in itself, which could have a positive effect on the antimicrobial activity of the extracts. The choice of the carrier plays an essential role in the antibacterial effectiveness of the propolis extract and the release kinetics of its active compounds. Gels made with a poloxamer base are a suitable form for incorporating propolis into skin preparations. Gels and bigels effectively release the active compounds of propolis, ensuring targeted antibacterial activity. Although oleogels did not exhibit antibacterial activity on their own, when integrated as a component in bigels, they enhance the lipid phase of the formulation, providing additional benefits for the skin, including softening the stratum corneum and nourishing the epidermis.

## Figures and Tables

**Table 1:** Composition of hydrogels, oleogels, and bigels with propolis DES extract (content in formula g/100 g).

Composition	G1	G2	G3	OG1	OG2	BG1	BG2
**Propolis DES**	10	10	10	10	10		
**P407**	13	15	18				
**Purified water**	ad 100	ad 100	ad 100				
**Castor oil**				ad 100			
**Olive oil**					ad 100		
**Beeswax**				0.5	1		
**0.1 M NaOH**	Qs pH 5.5–6.5	Qs pH 5.5–6.5	Qs pH 5.5–6.5				
**Appierance 200 µm**	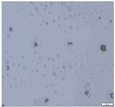	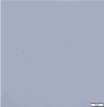	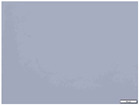		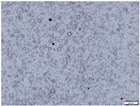	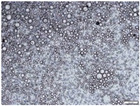	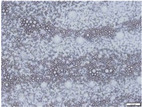

In the process of the production of bigels BG1 and BG2, the ratio between hydrogel and oleogel was 1:1 (G2:OG1; G2:OG2).

## Data Availability

The data presented in this study are openly available in the article.

## References

[1] Marsella R., Olivry T., Carlotti D.N. (2011). Current evidence of skin barrier dysfunction in human and canine atopic dermatitis. Vet. Dermatol..

[2] Hill P. (2002). Small Animal Dermatology: A Practical Guide to the Diagnosis and Managment of Skin Diseases in Dogs and Cats.

[3] Olivry T., Mueller R.S. (2003). International task force on canine atopic dermatitis. Evidence-based veterinary dermatology: A systematic review of the pharmacotherapy of canine atopic dermatitis. Vet. Dermatol..

[4] Hillier A., Griffin C.E. (2001). The ACVD task force on canine atopic dermatitis (I): Incidence and prevalence. Vet. Immunol. Immunopathol..

[5] Watson T.D.G. (1998). Diet and skin disease in dogs and cats. J. Nutr..

[6] Miller W.H., Griffin C.E., Campbell K.L. (2013). Muller & Kirk’s Small Animal Dermatology.

[7] Hillier A., Lloyd D.H., Weese J.S., Blondeau J.M., Boothe D., Breitschwerdt E., Guardabassi L., Papich M.G., Rankin S., Turnidge J.D. (2014). Guidelines for the diagnosis and antimicrobial therapy of canine superficial bacterial folliculitis (Antimicrobial Guidelines Working Group of the International Society for Companion Animal Infectious Diseases). Vet. Dermatol..

[8] Guardabassi L., Houser G.A., Frank L.A., Papich M.G., Guardabassi L., Jensen L.B., Kruse H. (2008). Guidelines for antimicrobial use in dogs and cats. Guide to Antimicrobial Use in Animals.

[9] Paul N.C., Damborg P., Guardabassi L. (2015). Dam-to-offspring transmission and persistence of *Staphylococcus pseudintermedius* clones within dog families. Vet. Dermatol..

[10] Lai C.-H., Ma Y.-C., Shia W.-Y., Hsieh Y.-L., Wang C.-M. (2022). Risk Factors for Antimicrobial Resistance of *Staphylococcus* Species Isolated from Dogs with Superficial Pyoderma and Their Owners. Vet. Sci..

[11] Štempelová L., Kubašová I., Bujňáková D., Karahutová L., Gálová J., Kužma E., Strompfová V. (2023). Antimicrobial activity of organic acids against canine skin bacteria. Vet. Res. Commun..

[12] Zhao L., Chen J., Bai B., Song G., Zhang J., Yu H., Huang S., Wang Z., Lu G. (2024). Topical drug delivery strategies for enhancing drug effectiveness by skin barriers, drug delivery systems and individualized dosing. Front. Pharmacol..

[13] Bertelloni F., Cagnoli G., Ebani V.V. (2021). Virulence and Antimicrobial Resistance in Canine *Staphylococcus* spp. Isolates. Microorganisms.

[14] Svetikiene D., Zamokas G., Jokubaite M., Marksa M., Ivanauskas L., Babickaite L., Ramanauskiene K. (2024). The Comparative Study of the Antioxidant and Antibacterial Effects of Propolis Extracts in Veterinary Medicine. Vet. Sci..

[15] Jenny J.C., Kuś P.M., Szweda P. (2024). Investigation of antifungal and antibacterial potential of green extracts of propolis. Sci. Rep..

[16] Samui T., Goldenisky D., Rosen-Kligvasser J., Davidovich-Pinhas M. (2021). The development and characterization of novel in-situ bigel formulation. Food Hydrocoll..

[17] Fan R., Cheng Y., Wang R., Zhang T., Zhang H., Li J., Song S., Zheng A. (2022). Thermosensitive Hydrogels and Advances in Their Application in Disease Therapy. Polymers.

[18] Diaz-Salmeron R., Toussaint B., Huang N., Bourgeois Ducournau E., Alviset G., Goulay Dufaÿ S., Hillaireau H., Dufaÿ Wojcicki A., Boudy V. (2021). Mucoadhesive Poloxamer-Based Hydrogels for the Release of HP-β-CD-Complexed Dexamethasone in the Treatment of Buccal Diseases. Pharmaceutics.

[19] Frolova Y., Sarkisyan V., Sobolev R., Makarenko M., Semin M., Kochetkova A. (2022). The Influence of Edible Oils’ Composition on the Properties of Beeswax-Based Oleogels. Gels.

[20] Abu-Seida A.M. (2015). Effect of Propolis on Experimental Cutaneous Wound Healing in Dogs. Vet. Med. Int..

[21] Tresch M., Mevissen M., Ayrle H., Melzig M., Roosje P., Walkenhorst M. (2019). Medicinal plants as therapeutic options for topical treatment in canine dermatology? A systematic review. BMC Vet. Res..

[22] Reichling J., Fitzi J., Hellmann K., Wegener T., Bucher S., Saller R. (2004). Topical tea tree oil effective in canine localised pruritic dermatitis—A multi-centre randomised double-blind controlled clinical trial in the veterinary practice. Dtsch. Tierarztl. Wochenschr..

[23] Ang M., Assis D.S., Ramos L.D.P., Hasna A.A., Queiroz T.S.D., Lima N.D., Berretta A.A. (2022). Antimicrobial and antibiofilm effect of Brazilian green propolis aqueous extract against dental anaerobic bacteria. Molecules.

[24] Huang S., Zhang C.-P., Wang K., Li G.Q., Hu F.-L. (2014). Recent advances in the chemical composition of propolis. Molecules.

[25] Anjum S.I., Ullah A., Khan K.A., Attaullah M., Khan H., Ali H., Bashir M.A., Tahir M., Ansari M.J., Ghramh H.A. (2019). Composition and functional properties of propolis (bee glue): A review. Saudi J. Biol. Sci..

[26] Da Cunha M.G., Franchin M., Galvão L., de Ruiz A., de Carvalho J.E., Ikegaki M., de Alencar S.M., Koo H., Rosalen P.L. (2013). Antimicrobial and antiproliferative activities of stingless bee *Melipona scutellaris* geopropolis. BMC Complement. Altern. Med..

[27] Stanciauskaitė M., Marksa M., Liaudanskas M., Ivanauskas L., Ivaskienė M., Ramanauskienė K. (2021). Poplar buds (*Populus balsamifera* L., *Populus nigra* L.) and Lithuanian propolis extracts: Comparison of their composition and biological activity. Plants.

[28] Woźniak M., Sip A., Mrówczyńska L., Broniarczyk J., Waśkiewicz A., Ratajczak I. (2022). Biological Activity and Chemical Composition of Propolis from Various Regions of Poland. Molecules.

[29] Barreto G.d.A., Cerqueira J.C., Reis J.H.d.O., Hodel K.V.S., Gama L.A., Anjos J.P., Minafra-Rezende C.S., Andrade L.N., Amaral R.G., Pessoa C.d.Ó. (2022). Evaluation of the potential of Brazilian red propolis extracts: An analysis of the chemical composition and biological properties. Appl. Sci..

[30] Ahmed E.T., Abo-Salem O.M., Osman A. (2011). The influence of Egyptian propolis on induced burn wound healing in diabetic rats; antibacterial mechanism. Sci. J. Med. Clin. Trials.

[31] Neuman M.G., Haber J.A., Malkiewicz I.M., Cameron R.G., Katz G.G., Shear N.H. (2002). Ethanol signals for apoptosis in cultured skin cells. Alcohol.

[32] Ranzer M.J., Chen L., DiPietro L.A. (2011). Fibroblast function and wound breaking strength is impaired by acute ethanol intoxication. Alcohol Clin. Exp. Res..

[33] Maxwell E.A., Bennett R.A., Mitchell M.A. (2018). Efficacy of application of an alcohol-based antiseptic hand rub or a 2% chlorhexidine gluconate scrub for immediate reduction of the bacterial population on the skin of dogs. Am. J. Vet. Res..

[34] Yu T., Yang L., Shang X., Bian S. (2024). Recovery of Cembratrien-Diols from Waste Tobacco (*Nicotiana tabacum* L.) Flowers by Microwave-Assisted Deep Eutectic Solvent Extraction: Optimization, Separation, and In Vitro Bioactivity. Molecules.

[35] Bobinaitė R., Viškelis P., Venskutonis P.R. (2012). Variation of total phenolics, anthocyanins, ellagic acid and radical scavenging capacity in various raspberry (*Rubus* spp.) cultivars. Food Chem..

[36] Babickaite L., Ramanauskiene K., Grigonis A., Ivaskiene M., Daunoras G., Klimiene I., Matusevicius A.P. (2016). Determination of antimicrobial activity of chlorhexidine gel. Acta Pol. Pharm..

[37] Uchil R.R., Kohli G.S., Katekhaye V.M., Swami O.C. (2014). Strategies to combat antimicrobial resistance. J. Clin. Diagn. Res..

[38] Hobson C., Chan A.N., Wright G.D. (2021). The Antibiotic Resistome: A Guide for the Discovery of Natural Products as Antimicrobial Agents. Chem. Rev..

[39] Kubkomawa H.I., Nafarnda D.W., Tizhe M.A., Daniel T.K., Shua N.J., Ugwu C.C., Ugwu C.C., Opara M.N., Neils J.S., Okoli I.C. (2020). Ethno-veterinary health management practices amongst livestock producers in Africa: A review. Adv. Agric. Sci..

[40] Wynn S., Fougère B., Wynn S., Fougère B. (2006). Clinical practice: Getting started. Veterinary Herbal Medicine.

[41] Olivry T., DeBoer D.J., Favrot C., Jackson H.A., Mueller R.S., Nuttall T., Prélaud P. (2015). International Committee on Allergic Diseases of Animals Treatment of canine atopic dermatitis: 2015 updated guidelines from the International Committee on Allergic Diseases of Animals (ICADA). BMC Vet. Res..

[42] Olivry T., Bizikova P. (2013). A systematic review of randomized controlled trials for prevention or treatment of atopic dermatitis in dogs: 2008–2011 update. Vet. Dermatol..

[43] Di Cerbo A., Morales-Medina J.C., Palmieri B., Pezzuto F., Cocco R., Flores G., Iannitti T. (2017). Functional foods in pet nutrition: Focus on dogs and cats. Res. Vet. Sci..

[44] Di Cerbo A., Palmieri B., Canello S., Guidetti G., Iannitti T. (2014). Functional foods in pets and humans. Int. J. Appl. Res. Vet. Med..

[45] Mazzeranghi F., Zanotti C., Di Cerbo A., Verstegen J.P., Cocco R., Guidetti G., Canello S. (2017). Clinical efficacy of nutraceutical diet for cats with clinical signs of cutaneus adverse food reaction (CAFR). Pol. J. Vet. Sci..

[46] Balcão V.M., Belline B.G., Silva E.C., Almeida P.F.F.B., Baldo D., Amorim L.R.P., Oliveira Júnior J.M., Vila M.M.D.C., Del Fiol F.S. (2022). Isolation and Molecular Characterization of Two Novel Lytic Bacteriophages for the Biocontrol of *Escherichia coli* in Uterine Infections: In Vitro and Ex Vivo Preliminary Studies in Veterinary Medicine. Pharmaceutics.

[47] Meroni G., Cardin E., Rendina C., Millar V.R.H., Filipe J.F.S., Martino P.A. (2020). In Vitro Efficacy of Essential Oils from Melaleuca Alternifolia and Rosmarinus Officinalis, Manuka Honey-Based Gel, and Propolis as Antibacterial Agents against Canine *Staphylococcus pseudintermedius* Strains. Antibiotics.

[48] Romero B., Susperregui J., Sahagún A.M., Diez M.J., Fernández N., García J.J., López C., Sierra M., Díez R. (2022). Use of medicinal plants by veterinary practitioners in Spain: A cross-sectional survey. Front. Vet. Sci..

[49] Cui H., Zhang C., Su K., Fan T., Chen L., Yang Z., Zhang M., Li J., Zhang Y., Liu J. (2024). Oregano Essential Oil in Livestock and Veterinary Medicine. Animals.

[50] Ebani V.V., Mancianti F. (2020). Use of Essential Oils in Veterinary Medicine to Combat Bacterial and Fungal Infections. Vet. Sci..

[51] Taheri M., Amiri-Farahani L. (2021). Anti-Inflammatory and Restorative Effects of Olives in Topical Application. Dermatol. Res. Pract..

[52] Malik N.S.A., Bradford J.M. (2006). Changes in oleuropein levels during differentiation and development of floral buds in “Arbequina” olives. Sci. Hortic..

[53] Di Pierro F. (2014). Roles of chemical complexity and evolutionary theory in some hepatic and intestinal enzymatic systems in chemical reproducibility and clinical efficiency of herbal derivatives. Sci. World J..

[54] Karaçam Z., Eroğlu K. (2003). Effects of episiotomy on bonding and mothers’ health. J. Adv. Nurs..

[55] Martinez-Lapiscina E.H., Clavero P., Toledo E., Julian B.S., Sanchez-Tainta A., Corella D., Lamuela-Raventos R.M., Martinez J.A., Martinez-Gonzalez M.Á. (2013). Virgin olive oil supplementation and long-term cognition: The Predimed-Navarra randomized, trial. J. Nutr. Health Aging.

[56] Gorzynik-Debicka M., Przychodzen P., Cappello F., Kuban-Jankowska A., Marino Gammazza A., Knap N., Wozniak M., Gorska-Ponikowska M. (2018). Potential health benefits of olive oil and plant polyphenols. Int. J. Mol. Sci..

[57] Radošević K., Ćurko N., Gaurina Srček V., Cvjetko Bubalo M., Tomašević M., Kovačević Ganić K., Radojčić Redovniković I. (2016). Natural Deep Eutectic Solvents as Beneficial Extractants for Enhancement of Plant Extracts Bioactivity. LWT Food Sci. Technol..

[58] Dumortier G., Grossiord J.L., Agnely F., Chaumeil J.C. (2006). A Review of Poloxamer 407 Pharmaceutical and Pharmacological Characteristics. Pharm. Res..

[59] Ibrahim N.A., Nada A.A., Eid B.M. (2018). Polysaccharide-Based Polymer Gels and Their Potential Applications. Polymer Gels.

[60] Verma A., Singh S., Kaur R.P., Jain U.K. (2013). Topical Gels as Drug Delivery Systems: A Review. Int. J. Pharm. Sci. Rev. Res..

[61] Venkataramani D., Tsulaia A., Amin S. (2020). Fundamentals and applications of particle stabilized emulsions in cosmetic formulations. Adv. Colloid. Interface Sci..

[62] Zhuang X., Clark S., Acevedo N. (2021). Bigels—Oleocolloid matrices—As probiotic protective systems in yogurt. J. Food Sci..

[63] Betancourt N., García-Contreras L., Sánchez T. (2015). Propolis in Dogs: Clinical Experiences and Perspectives (A Brief Review). Open J. Vet. Med..

[64] Toro-Vazquez J.F., Mauricio-Pérez R., González-Chávez M.M., Sánchez-Becerril M., Ornelas-Paz J.D.J., Pérez-Martínez J.D. (2013). Physical properties of organogels and water in oil emulsions structured by mixtures of candelilla wax and monoglycerides. Food Res. Int..

[65] Zilius M., Ramanauskienė K., Briedis V. (2013). Release of propolis phenolic acids from semisolid formulations and their penetration into the human skin in vitro. Evid. Based Complement. Altern. Med..

[66] Shaikh H.M., Anis A., Poulose A.M., Madhar N.A., Al-Zahrani S.M. (2022). Development of Bigels Based on Date Palm-Derived Cellulose Nanocrystal-Reinforced Guar Gum Hydrogel and Sesame Oil/Candelilla Wax Oleogel as Delivery Vehicles for Moxifloxacin. Gels.

[67] Viqhi A.V., Manggau M.A., Sartini S., Wahyudin E., Rahman L., Yulianti R., Permana A.D., Awal S.A. (2021). Development of Propolis (*Apis trigona*)-loaded Nanoemulgel for Improved Skin Penetration of Caffeic Acid: The Effect of Variation of Oleic Acid Concentration. Open Access Maced. J. Med. Sci..

[68] Freitas A.S., Cunha A., Oliveira R., Almeida-Aguiar C. (2022). Propolis antibacterial and antioxidant synergisms with gentamicin and honey. J. Appl. Microbiol..

[69] Pobiega K., Kraśniewska K., Derewiaka D., Gniewosz M. (2019). Comparison of the antimicrobial activity of propolis extracts obtained by means of various extraction methods. J. Food Sci. Technol..

[70] Kasparaviciene G., Maslii Y., Herbina N., Kazlauskiene D., Marksa M., Bernatoniene J. (2024). Development and Evaluation of Two-Phase Gel Formulations for Enhanced Delivery of Active Ingredients: Sodium Diclofenac and Camphor. Pharmaceutics.

[71] An S.-H., Ban E., Chung I.-Y., Cho Y.-H., Kim A. (2021). Antimicrobial Activities of Propolis in Poloxamer Based Topical Gels. Pharmaceutics.

[72] Alkarri S., Bin Saad H., Soliman M. (2024). On Antimicrobial Polymers: Development, Mechanism of Action, International Testing Procedures, and Applications. Polymers.

[73] Wang S., Chen K., Liu G. (2022). Monoglyceride oleogels for lipophilic bioactive delivery—Influence of self-assembled structures on stability and in vitro bioaccessibility of astaxanthin. Food Chem..

[74] Ferreira L.M.d.M.C., Cruz N.F.d., Lynch D.G., Costa P.F.d., Salgado C.G., Silva-Júnior J.O.C., Rossi A., Ribeiro-Costa R.M. (2024). Propolio turintis hidrogelis: Fizinė charakteristika ir biologinio aktyvumo įvertinimas, siekiant jį panaudoti odos pažeidimų gydymui. Pharmaceuticals.

[75] Kulawik-Pióro A., Miastkowska M. (2021). Polymeric Gels and Their Application in the Treatment of Psoriasis Vulgaris: A Review. Int. J. Mol. Sci..

[76] Ferreira L.M.d.M.C., Modesto Y.Y., Souza P.D.Q.d., Nascimento F.C.d.A., Pereira R.R., Converti A., Lynch D.G., Brasil D.d.S.B., da Silva E.O., Silva-Júnior J.O.C. (2024). Characterization, Biocompatibility and Antioxidant Activity of Hydrogels Containing Propolis Extract as an Alternative Treatment in Wound Healing. Pharmaceuticals.

